# Epithelioid Angiomyolipoma Masquerading as Renal Cell Carcinoma: A Case Report

**DOI:** 10.7759/cureus.108184

**Published:** 2026-05-03

**Authors:** Sailuja Maharjan, Divya V, Prakruthi Lokesh, Srivatsa N, Rekha V Kumar

**Affiliations:** 1 Tumor Pathology, Sri Shankara Cancer Hospital and Research Centre, Bengaluru, IND; 2 Uro-Oncology, Sri Shankara Cancer Hospital and Research Centre, Bengaluru, IND

**Keywords:** angiomyolipoma, epithelioid, renal cell carcinoma, tfe3, tsc

## Abstract

Epithelioid angiomyolipoma (EAML) is a rare variant of angiomyolipoma with malignant behavior characterized by predominantly epithelioid cells and minimal fat, mimicking renal cell carcinoma (RCC) on imaging. Preoperative diagnosis is challenging due to its non-specific clinical and radiological features, necessitating a histopathological examination for accurate diagnosis. Unlike classic angiomyolipoma, EAML can demonstrate aggressive behavior, including local invasion, recurrence, and distant metastasis. Owing to the rarity of the tumor, there is a paucity of data regarding the immunohistochemical markers and molecular profiles for predicting biomarkers in EAML. We report a case of renal EAML in a 23-year-old female with liver and lymph node metastases, which was preoperatively diagnosed as RCC. Histopathological examination and immunohistochemistry favored a diagnosis of EAML. Furthermore, next-generation sequencing confirmed a*TFE3* rearrangement with an *SFPQ* fusion. However, *TFE3* immunopositivity was also noted in our case, serving as a potential pitfall by raising suspicion for *TFE3-*rearranged RCC. Therefore, this case highlights the importance of a comprehensive evaluation integrating morphology, immunohistochemistry, and molecular studies for accurate diagnosis.

## Introduction

Renal angiomyolipoma (AML), a member of the perivascular epithelioid cell tumor (PEComa) family, is characterized by co-expression of smooth muscle and melanocytic markers and accounts for ~1% of renal tumors [1,2]. It is classified into classic AML and epithelioid AML (EAML) [3]. Classic AML is a benign mesenchymal tumor composed of a triphasic population of adipose tissue, smooth muscle, and thick-walled vessels [4,5].

EAML is a rare variant with malignant behavior, accounting for 4.6% of AMLs [6]. It is characterized by predominantly epithelioid cells (≥80%) and minimal fat (<5%), with extrarenal extension in up to one-third of cases [2,7-9]. Its clinical and radiological overlap with renal cell carcinoma (RCC) makes the diagnosis challenging, necessitating histopathology with immunohistochemistry (IHC) for confirmation [2,8-11].

We report a case of giant EAML (18 cm) with liver infiltration and lymph node metastases, preoperatively diagnosed as RCC. This case is significant due to the diagnostic challenge posed by its overlap with *TFE3-*rearranged RCC, particularly in view of *TFE3* immunopositivity. Furthermore, next-generation sequencing (NGS) revealed a *TFE3* rearrangement with *SFPQ* as the fusion partner. Therefore, our case highlights the importance of a comprehensive evaluation integrating morphology, immunohistochemistry, and molecular studies for accurate diagnosis owing to its initial misdiagnosis as RCC.

## Case presentation

A 23-year-old woman presented with abdominal pain, fever, and severe fatigue of one month duration, following a recent lower-segment cesarean section. There was no significant past or family history. Examination revealed mild pallor and right hypochondriac tenderness. Laboratory tests showed a hemoglobin of 9.6 g/dL (reference range: 12-15 g/dL), serum albumin of 3.1 g/dL (reference range: 3.5-5.2 g/dL), and leukocyte level of 12,000/cumm (reference range: 4,000-10,000/cumm), with otherwise normal renal and liver function.

**Figure 1:**
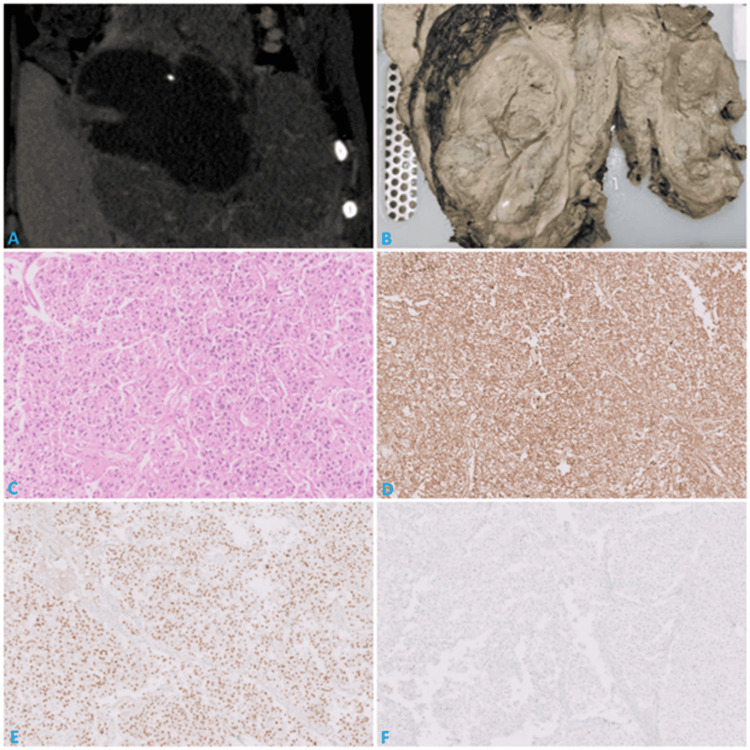
(A) PET-CT scan showing a right renal solid cystic mass with heterogeneous enhancement and necrosis, completely replacing the kidney with indentation of the liver. (B) Gross image of right nephrectomy showing a gray-white tumor with necrosis. (C) Histopathological examination showing predominantly epithelioid cells (10×). (D) IHC showing diffuse cytoplasmic HMB45 staining (10×). (E) IHC showing diffuse nuclear TFE3 staining (10×). (F) IHC showing no PAX8 staining (10×).

PET-CT demonstrated a large (14.5 × 10.8 × 20 cm) heterogeneous cortico-medullary phase-enhancing necrotic mass replacing the right kidney, with renal vein involvement and extension, suggestive of RCC with sarcomatoid features (Figure 1A). The mass abutted the liver with indistinct fat planes, and hyperdense areas suggested intratumoral hemorrhage. The paucity of fat components in EAML, in contrast to classic AML, makes the preoperative accurate diagnosis of EAML challenging, often leading to misdiagnosis as RCC.

Following emergency angioembolization, the patient underwent right radical nephrectomy with resection of liver segments V and VI and retroperitoneal lymph node dissection. Grossly, the tumor measured 18 × 12 × 13 cm, was lobulated, gray-white, and extensively necrotic (~70%), with invasion into renal sinus fat and liver parenchyma (Figure 1B).

Histology revealed a malignant tumor predominantly composed of epithelioid cells (>80%) arranged in nests and sheets, with thick-walled vessels and spindle cell areas. Tumor cells showed abundant eosinophilic to clear cytoplasm, nuclear pleomorphism, prominent nucleoli visible at 100×, and occasional multinucleated giant cells (Figure 1C). Mitotic activity was 2-3/2 mm². Tumor infiltration into the renal sinus, vessels, and liver, with metastasis in three retroperitoneal lymph nodes, was noted. These morphological features raised the possibility of RCC.

Immunohistochemistry showed diffuse positivity for HMB45, Melan-A, *CD68*, and *TFE3*, with focal SMA positivity, supporting a perivascular epithelioid cell origin (Figures 1D, 1E). Markers for epithelial tumors (panCK, EMA, PAX8, CD10, CAIX) and melanoma (S100) were negative (Figure 1F). Ki-67 index was ~25%.

Based on these findings, a diagnosis of epithelioid angiomyolipoma was made. NGS revealed a *TFE3* rearrangement with *SFPQ* as the fusion partner. The patient has been under Everolimus therapy with close follow-up and has been doing well for three months.

## Discussion

EAML is a rare and aggressive variant of angiomyolipoma, first described by Mai et al. in 1996 [7,8]. Unlike classic AML, which is a benign triphasic tumor composed of an admixture of adipose tissue, smooth muscle, and blood vessels, EAML is characterized by predominant epithelioid morphology with a minimal adipose component [2,6]. It demonstrates malignant potential, including invasive growth, recurrence, vascular invasion, and distant metastasis [1,4,5]. The liver is the most common metastatic site, followed by the lungs, lymph nodes, pelvis, and peritoneum [7,10,11]. To date, only 46 metastatic cases have been reported, including 17 with liver metastasis [8].

AML occurs across a wide age range, peaking in the third to fourth decades, with a female predominance, possibly related to estrogen and progesterone receptor expression, though some studies show no sex bias [3,7,10,12]. Most cases are sporadic; however, association with tuberous sclerosis (TSC) is well established, particularly in younger patients [1]. TSC is an autosomal dominant disorder caused by mutations in the tumor suppressor genes *TSC1* (tuberin, chromosome 9) and *TSC2* (hamartin, chromosome 16), resulting in dysregulation of the mTOR pathway and multisystem hamartomas [4,6,9,12,13]. Approximately 80% of TSC patients develop AML, whereas sporadic cases are frequently associated with *TSC2* mutations [8].

Most renal EAMLs are asymptomatic and incidentally detected, though some present with abdominal pain, hematuria, or a palpable mass [2,3,12]. Preoperative diagnosis is challenging due to nonspecific clinical and radiologic features [1,10]. The minimal fat content often results in hyperdense lesions on imaging, frequently leading to misdiagnosis as RCC [5,13]. Consequently, a definitive diagnosis is typically established on postoperative histopathology, while biopsy interpretation remains difficult due to overlap with entities such as clear cell RCC (eosinophilic variant), MiTF translocation RCC, and chromophobe RCC, necessitating immunohistochemistry [1,4,6,7,9].

According to WHO criteria, the diagnosis of EAML requires ≥80% epithelioid cells [2,3,14]. Two histologic patterns are described: a carcinoma-like pattern with large atypical eosinophilic cells in nests separated by vascular septa and a diffuse pattern composed of epithelioid and plump spindle cells [5,12]. Our case showed >80% epithelioid cells with thick-walled vessels and spindle cell areas, without adipocytic differentiation.

Tumor cells in EAML typically express melanocytic markers (HMB45, Melan-A, SOX10, and cathepsin K) and myogenic markers (SMA) [10,13]. Negative staining for panCK, EMA, and PAX8 helps exclude epithelial and renal tubular tumors, while the absence of S100 rules out melanoma [4,6,8]. In this case, overlap with *TFE3*-rearranged RCC was noted due to *TFE3* positivity; however, co-expression of SMA and CD68, along with PAX8 negativity, supported EAML, highlighting the importance of a comprehensive panel of markers to arrive at a definitive diagnosis. Nevertheless, the characteristic morphological features of *TFE3-*rearranged RCC, such as predominantly clear cells, papillary structures, and psammoma bodies, were lacking in our case, emphasizing the importance of correlating immunohistochemical findings with morphology.

The rarity of EAML limits accurate prediction of malignant behavior; however, several clinicopathological criteria have been proposed. Lei et al. suggested that the presence of ≥3 of the following features indicates malignancy: necrosis, tumor size >9 cm, venous tumor thrombus, and ≥70% epithelioid cells [2,3]. All these features were present in our case, supporting its aggressive nature. The proportion of epithelioid cells also correlates with progression risk (<10%: none; 80-95%: low; >95%: high) [1,15]. Additional adverse prognostic factors include concurrent tuberous sclerosis or classic AML, tumor size >7 cm, extrarenal extension, renal vein involvement, necrosis, and carcinoma-like histology [7,14]. Emerging molecular data suggest alterations in *TP53*, *ATRX*, *APC*, *NF1*, *TERT*, *TFE3*, and *RB1* may further stratify high-risk cases and provide insights into the molecular landscape of EAML [5,6]. NGS identified a *TFE3* rearrangement with *SFPQ* as the fusion partner in our case. This finding is comparable to the study by Sangoi et al., in which 35% of cases demonstrated *TFE3* alterations, all involving *TFE3-SFPQ* rearrangement, but mutation in the TSC/MTOR pathway was more prevalent, occurring twice as frequently as *TFE3* rearrangement [4]. Unlike classic angiomyolipoma, which expresses both melanocytic and smooth muscle markers, *TFE3-*rearranged epithelioid angiomyolipomas are found to have a specific immunophenotype with expression of a melanocytic marker (HMB45) and minimal/absent desmin or actin, which can serve as a surrogate IHC marker in resource-limited settings where molecular testing is not feasible [16].

Surgical resection remains the mainstay of treatment [7,15]. Adjunctive options include angioembolization and ablative therapies [1]. Immune checkpoint inhibitors (PD-L1-targeted) have shown therapeutic benefit, while *TFE3-*rearranged PEComas may respond to VEGF/VEGFR-targeted therapy, underscoring the importance of molecular testing [8]. Additionally, chemotherapeutic agents such as doxorubicin and mTOR inhibitors (everolimus, sirolimus) have demonstrated efficacy in metastatic disease [2,13]. Long-term surveillance is essential for early detection of recurrence and metastasis.

## Conclusions

Epithelioid angiomyolipomas are rare tumors with malignant potential and a propensity for recurrence and metastasis, necessitating long-term surveillance. Their nonspecific clinical and radiologic features make diagnosis challenging, and confirmation relies on histopathology with immunohistochemistry as the gold standard. Additionally, molecular studies can be valuable in *TFE3-*rearranged EAML, given its potential therapeutic implications.
